# Zishen pingchan granules combined with pramipexole in the improvement of depressive symptoms in Parkinson's disease: a prospective, multicenter, randomized, double-blind, controlled clinical study

**DOI:** 10.1186/s12967-022-03551-z

**Published:** 2022-08-12

**Authors:** Houxu Ning, Hao Zhou, Jingru Ren, Gaiyan Zhou, Ning Yang, Zhenfu Wang, Canxing Yuan, Zuojun Tian, Juping Chen, Lihua Shen, Huifen Zheng, Yang Zhao, Haidong Wang, Weiguo Liu, Zhenguo Liu

**Affiliations:** 1 Department of Chinese Medicine, The Affiliated Brain Hospital of Nanjing Medical University, Nanjing, 210029 China; 2 Department of Neurology, The Affiliated Brain Hospital of Nanjing Medical University, Nanjing, 210029 China; 3 Department of Neurology, Chinese PLA General Hospital, Beijing, 100036 China; 4 Department of Neurology, Xinhua Hospital, Shanghai Jiao Tong University School of Medicine, Shanghai, 200092 China; 5 Department of Neurology, Longhua Hospital, Shanghai University of Traditional Chinese Medicine, Shanghai, 200032 China; 6 Department of Neurology, the First Affiliated Hospital of Guangzhou Medical University, Guangzhou Medical University, Guangzhou, 510000 China; 7 Department of Neurology, Changshu Hospital of Traditional Chinese Medicine, Changshu, 215500 China; 8 Department of Neurology, Affiliated Hospital of Nantong University, Nantong University, Nantong, 226000 China; 9 Department of Neurology, Geriatric Hospital of Nanjing Medical University, Nanjing Medical University, Nanjing, 210000 China; 10 Department of Neurology, Nanjing Hospital of Chinese Medicine, Nanjing University of Chinese Medicine, Nanjing, 210022 China

**Keywords:** Depression in the Parkinson's disease, Zishen Pingchan granules, Pramipexole, Randomized controlled trial

## Abstract

**Background and objective:**

Zishen Pingchan granule (ZPG), a traditional Chinese herbal recipe for treating Parkinson’s disease (PD), is usually used as an add-on drug with some antiparkinsonian drugs in China. The objectives of this study were to evaluate the efficacy, safety, and tolerability of ZPG combined with pramipexole in the treatment of depression in PD (dPD).

**Methods:**

A 12-week, multicenter, randomized, double-blind, and placebo-controlled study on ZPG was performed on a total of 200 patients who were treated with pramipexole but still had mild to moderate depressive symptoms. Patients were randomly divided into ZPG (*n* = 100) or placebo (*n* = 100). The primary effective result was the mean change from the baseline on the Hamilton Depression Scale 17 items (HAM-D-17) over 12 weeks and the clinical efficacy rate. Secondary endpoints were the mean change from the baseline in the Geriatric Depression Scale (GDS-15), Unified Parkinson's disease rating scale Part III (UPDRS III), Parkinson's quality of life scale (PDQ-8), and Parkinson's disease sleep scale (PDSS-2) over 12 weeks.

**Results:**

After 12 weeks of treatment, ZPG significantly reduced the mean [95% confidence interval] HAMD score vs. placebo (− 1.43 scores [− 2.50, − 0.36]; *p* = 0.009). The clinical remission rate and responders of the ZPG group were higher than those of the placebo (46.1% vs. 31.0%; *p* = 0.041; 34.8% vs. 18.4%; *p* = 0.014). A significant improvement in the PDSS-2 score was also observed in the ZPG group compared with that in the placebo group (− 3.56 scores [− 5.77, − 1.35]; *p* = 0.002). A total of 7 patients (7.1%) in the ZPG group had mild adverse events (AEs) vs 9 patients (9%) in the placebo group. No severe AEs were observed in either group. The randomization and controlled clinical study revealed that ZPG was effective, safe, and well-tolerated.

**Conclusion:**

ZPG combined with pramipexole further reduced the depressive symptoms and improved the sleeping quality of PD patients.

*Trial registration* The protocol was retrospectively registered at the Chinese Clinical Trial Registry, Unique identifier: ChiCTR1800019942, date of registration: December 9, 2018; http://www.chictr.org.cn/showproj.aspx?proj=30432

## Introduction

Parkinson’s disease (PD) is a progressive neurodegenerative disorder characterized by tremor, rigidity, and bradykinesia [1]. PD also induces a long list of non-motor symptoms, particularly depression, in addition to the motor manifestations [2]. According to previous studies, the prevalence rates of depression in PD (dPD) range from 2.7 to 90% [3]. Long-term depression aggravates motor symptoms and is closely related to a decline in the quality of life, cognitive impairment, higher levels of care dependency, and increased caregiver distress [4], which deserves also attention in the treatment of dPD.

Several previous large randomized controlled trials investigated the efficacy of pramipexole (PPX) in patients with dPD. All the results indicate that PPX is effective in improving the depressive symptoms in these patients [5–7]. Hence, the Movement Disorders Society recommends PPX as the first-line drug to treat dPD [8]. Unfortunately, some patients do not respond well to PPX and require additional medication.

Zishen Pingchan granule (ZPG) is a traditional Chinese herbal recipe, widely used to treat PD for over 30 years[9]. ZPG protects nerve cells by inhibiting the hyperactivation of extracellular signal-regulated kinase and c-Jun N-terminal kinase pathways to reduce the inflammatory reaction [10]. A recent randomized, double-blind, placebo-controlled trial showed that the treatment with ZPG significantly improves the depressive symptoms of PD, with quite noticeable improvements in dyskinesia and delay in the progression of the disease [9].

To the best of our knowledge, the studies combining ZPG and PPX to improve the depressive symptoms in dPD patients have not been performed. Therefore, the objective of this study is to determine the clinical efficacy, safety, and tolerability of ZPG as an additional therapy for PD patients whose depressive symptoms were not optimally controlled by PPX.

## Patients and methods

This 12-week, prospective, randomized, double-blind, placebo-controlled trial on ZPG as an add-on drug to PPX therapy in PPX-treated patients with mild to moderate depressive symptoms was conducted at 9 hospitals in Jiangsu, Beijing, Shanghai, and Guangdong, China. The trial protocol was produced according to the *Declaration of Helsinki* and *Good Clinical Practice guidelines* [11, 12], with the approval of the Ethics Committee of each research unit with the following ethic code: ChiCTR1800019942. The study was registered at www.chictr.org.cn before the enrollment of the first patient. All participants provided written informed consent for all procedures in this study.

### Participants

Participants were enrolled from March 2019 to May 2021. The inclusion criteria were the following: (1) Patients aged ≥ 40 and ≤ 80 years old, Chinese speaking in both genders meeting the Movement Disorder Society Clinical Diagnostic Criteria for PD [13]. (2) Mild-to-moderate depression, meeting the diagnostic criteria of depression referred to *the Diagnostic and Statistical Manual of Mental Disorders (5th Edition)*, with the score of 17 items on the Hamilton Depression scale (HAM-D-17) ≥ 8 and ≤ 24 [14] (3) The dosage of PPX was ≥ 0.75 mg/day in the past four weeks. (4) Hoehn-Yahr grade ≤ 4.

The exclusion criteria were the following: (1) Major depression. (2) Medical history of cerebrovascular disease, encephalitis, poisoning, drug-induced parkinsonism, vascular parkinsonism, or atypical forms of parkinsonism. (3) Severe heart, lung, and kidney disease. (4) Pregnant or lactating women. (5) Participation in other clinical trials at the same time.

### Study design

Patients underwent screening and baseline assessments at week 0, and those who met the eligibility criteria were randomized 1:1 by computer for the addition of ZPG or matching placebo. Subsequent study visits were performed at week 2, 6, and 12. Dosages of PD medications, including dopamine agonists and any others were unchanged throughout the entire study.

### Sample size calculation, randomization, and masking

A 5% significance level was considered; thus, the α value was determined at 0.05 and theβ value was 0.10. The Z_α/2_ value was 1.96, based on the Z value table for the two-tailed distribution. The Z_β_ value was based on the Z value table for a one-tailed distribution of 1.28. The minimum necessary sample size was determined by the following formula:

$$n1=n2=\frac{{\left[{Z}_{\alpha /2}\sqrt{2\overline{P}(1-\overline{P})}+{Z}_{\beta }\sqrt{{\mathrm{P}}_{1}\left(1-{\mathrm{P}}_{1}\right)+{\mathrm{P}}_{2}\left(1-{\mathrm{P}}_{2}\right)}\right]}^{2}}{{({\mathrm{P}}_{1}-{\mathrm{P}}_{2})}^{2}}\approx 80$$

In this equation, n is the sample size of each group. P_1_ is the improvement rate of the depressive symptoms in the treatment group and P_2_ is the improvement rate of the placebo group. Based on previous literature [10], our estimation was P_1_ = 0.7 and P_2_ = 0.45. According to the dropout rate of 20%, a sample of 200 subjects (100 per group) was required.

The randomization code was generated by SAS 9.3 with a block size of four to provide a balanced distribution of the treatment groups within each center. To preserve masking, access to the randomization code was restricted to biostatistics experts and pharmaceutical personnel who generated the code and labeled and packaged the study drugs. Investigators, clinical monitors, and patients were masked to the identity of the treatment allocation.

### Study medication

During the double-blind 12 weeks of medication, the patients were randomly allocated into 2 groups: (1) Zishen Pingchan group: 1 pack ZPG (6.75 g), 2 times per day. PPX that patients were already taken was kept; (2) placebo group: 1 pack ZPG simulation agent, 2 times per day. PPX that patients were already taken was kept. Both ZPG and placebo (batch number:190101) were produced by Sichuan New GreenMedicine Science and Technology Development Co. Ltd., Chendu, 611900 China. PPX was produced by Shanghai Boehringer Ingelheim Pharmaceutical Co., Ltd. The placebo contained 10% of the active ingredient of ZPG and had an identical taste and appearance to the experimental drugs to preserve blinding. All subjects should not take other antidepressants during the entire study period.

ZPG is a Chinese herbal medicine approved by the China National Medical Products Administration for dPD, and it is composed of 12 herbs: Radix Rehmanniae preparata (shú dì huáng), Lycium barbarum (gŏu qĭ zĭ), Morus parasitic (Sāng jì shēng), Rhizoma Gastrodiae (tiān má), Bombyx Batryticatus (Jiāng cán), Curcumae Rhizoma (É zhú), Paeoniae Radix Alba (Bái sháo), Arisaematis Rhizoma (Tiān nán xīng), Rhizoma anemarrhenae (Zhī mǔ), Lilii Bulbus (Bǎi hé), Acori Tatarinowii Rhizoma(Shí chāng pú), Polygala tenuifolia Willd (Yuǎn zhì).

### Efficacy measurements

The patients' condition was evaluated at baseline and at 2, 6, and 12 weeks after treatment. All scales were assessed in the “on” period. The measure of the primary outcome was the change in the HAM-D score from the baseline to week 12 and the additional primary outcome was the clinical efficacy. The clinical efficacy was evaluated according to the reduction rate of the HAM-D score from the baseline to week 12 calculated using the following formula: HAM-D score reduction rate from the baseline to week 12 = [(HAM-D point at the baseline—HAM-D point at week 12)/ HAM-D point at baseline] × 100%. Prespecified dichotomous HAM-D outcomes were also assessed, including clinical remission, characterized by a HAM-D score < 8 at week 12, and responders, characterized by a ≥ 50% reduction in HAM-D score from the baseline to week 12.

The secondary outcomes included the mean change in scores on the Geriatric Depression Scale (GDS-15), Unified Parkinson’s Disease Rating Scale part III (UPDRS III), Parkinson’s Patient Quality of Life Scale (PDQ-8), and Parkinson’s Disease Patient Sleep Quality Scale (PDSS-2) from the baseline to week 12.

### Safety assessment

The safety assessments was as follows: (1) The laboratory parameters included the complete blood count, as well as hepatic and renal function; (2) electrocardiography; (3) occurrence of adverse events (AEs) during this study.

### Statistical analysis

Statistical analysis was performed using SPSS version 26.0. The Kolmogorov–Smirnov test was used to assess the normality of the data. Differences in gender and H-Y stage between groups were assessed using the chi-square test. Differences in baseline demographic and clinical variables, excluding gender and H-Y stage, between patients were assessed using a two-sample *t*-test when the data were normally distributed; otherwise, the Mann–Whitney U test was used.

The primary efficacy outcome was the mean change in HAM-D score from the baseline to week 12, comparing the ZPG and placebo group. The primary efficacy analysis was performed using the modified intent-to-treat (mITT) population (defined as all randomized subjects taking at least one dose of the study drug and having a baseline and at least one post-baseline efficacy assessment) and used a repeated measures model with week in the experiment; the treatment was included as a fixed effect and the baseline HAMD score as a covariate. The last observation carried forward analysis was used for data lost at endpoints. Additional primary efficacy outcome variables included the rates of “remission” and “responders” at week 12. The chi-square test was used to compare the distribution of subjects between groups. Safety results were assessed using a safety population that included all subjects who received at least one dose of the study drug.

Secondary efficacy outcomes included the change in UPDRS III, GDS-15, PDQ-8, and PDSS-2 scores from the baseline to week 12, which were analyzed in the same way as the primary outcome variable. A value of *p* < 0.05 was considered statistically significant.

## Results

### Subject arrangement

**Fig. 1:**
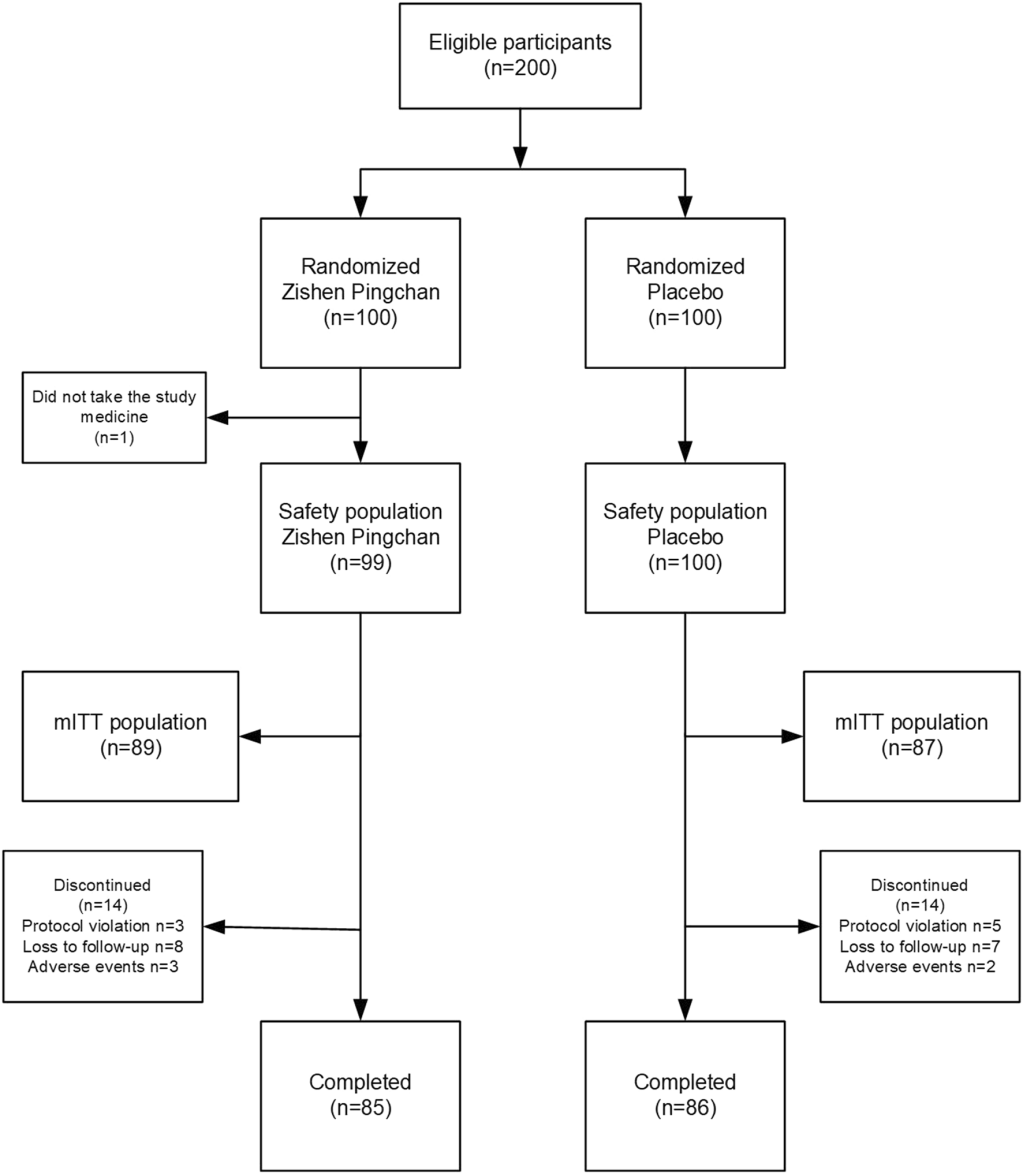
Trial profile

Two hundred subjects were enrolled and randomly divided in this study (Fig. 1). One subject of the Zishen Pingchan group was enrolled and randomized but not dosed and was therefore not part of the Safety population (defined as having taken at least one dose of the study drug) or mITT population. The Safety population was composed of 199 subjects (99.5%), the mITT was composed of 176 (88%) subjects, and 171 (85.5%) completed the study.

### Demographics and baseline characteristics

Subject demographics and baseline PD characteristic of the mITT population are listed in Table 1. The mean age ± SD was 68.1 ± 8.0 years in the ZPG group *vs* 66.4 ± 9.8 years in the placebo group, and 42 (47.7%) in the ZPG group were male *vs* 45 (51.1%) in the placebo group. The mean duration of PD was 92.4 ± 48.1 months in the ZPG group *vs* 80.8 ± 46.9 months in the placebo group, and the median Mini-mental State Examination (MMSE) scores were 29 (27.30) in both groups. Mean UPDRS-III scores ± SD was 23.98 ± 11.86 in the ZPG group *vs* 23.93 ± 11.14 in the placebo group, and mean LED ± SD was 246.38 ± 123.90 mg/day in the ZPG *vs* 234.40 ± 141.63 mg/day in the placebo group. No significant difference in demographics and baseline characteristics was observed between groups.

**Table 1:** Patient demographics and baseline characteristics (mITT population)

Parameter	Zishen Pingchan (n = 89)	Placebo (n = 87)	*p*-value
Male gender; n (%)	42 (47.7%)	45 (51.1%)	0.651^b^
Age; years mean ± SD	68.1 ± 8.0	66.4 ± 9.8	0.203^a^
Duration of PD; months mean ± SD	92.4 ± 48.1	80.8 ± 46.9	0.196^a^
MMSE score; median (IQR)	29 (27, 30)	29 (27, 30)	0.765^c^
UPDRS-III; mean ± SD	23.98 ± 11.86	23.93 ± 11.14	0.979^a^
LED; mean ± SD	246.38 ± 123.90	234.40 ± 141.63	0.224^a^
*Hoehn-Yahr stage; n (%)*			0.949^b^
Stage 1 Stage 1.5 Stage 2 Stage 2.5 Stage 3 Stage 4	11 (12.4%) 5 (5.6%) 43 (48.3%) 9 (10.1%) 20 (22.5%) 1 (1.1%)	8 (9.2%) 7 (8.0%) 42 (48.3%) 9 (10.3%) 19 (21.8%) 2 (2.3%)	

Parametric variables are presented as mean ± SD, non-parametric variables are presented as median (interquartile range) and categorical variables are presented as n (%)

PD, Parkinson’s disease; UPDRS, Unified Parkinson Disease Rating Scale; MMSE, Mini-mental State Examination; SD, standard deviation; IQR, interquartile range; LED: Levodopa equivalent dose

^a^Two-sample *t*-test

^b^Chi-squared test

^c^Mann-Whitney U test

### Efficacy

**Fig. 2:**
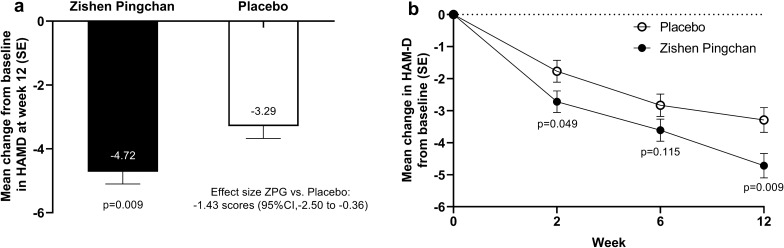
Change of the HAMD score from the baseline. **a** Primary endpoint: Change in the least-squares (LS) mean ± SE HAMD. (mITT population, repeated measure analysis of the covariance model, last observation carried forward). **b** Mean change of the HAMD from the baseline (mITT population, repeated measure analysis of the covariance model, last observation carried forward). SE, standard error

The results showed a significant improvement in the HAM-D scores from the baseline to week 12 in the ZPG group compared to the placebo group (least-squares [LS] mean difference ± SE, − 1.43 ± 0.54; 95% confidence interval [CI], − 2.50, − 0.36; *p* = 0.009) (Fig. 2). HAM-D in the ZPG group improved from 13.00 ± 4.24 to 8.39 ± 4.02 (LS mean ± SE treatment effect, − 4.72 ± 0.38). HAMD in the placebo group improved from 13.48 ± 3.97 to 10.08 ± 4.38 (treatment effect, − 3.29 ± 0.39) (Table 2). The additional primary outcome showed that the Clinical remission rate and responders in the ZPG group were significantly higher than those in the placebo group (remission: 46.1% in the ZPG group *vs*. 31.0% in the placebo group; responders: 34.8% in the ZPG group *vs*. 18.4% in the placebo group) (Table 2).

**Table 2:** Efficacy outcomes (mITT population)

	Zishen Pingchan (n = 89)	Placebo (n = 87)	*p*-value Treatment effect (Zishen Pingchan-placebo)
*HAMD score*			
Baseline; mean ± SD	13.00 ± 4.24	13.48 ± 3.97	
Week 12; mean ± SD	8.39 ± 4.02	10.08 ± 4.38	
Treatment effect; LS mean ± SE (95%CI)	− 4.72 ± 0.38 (− 5.47, − 3.97)	− 3.29 ± 0.39 (− 4.05, − 2.53)	0.009^a*^
*UPDRS-III score*			
Baseline; mean ± SD	23.98 ± 11.86	23.93 ± 11.14	
Week 12; mean ± SD	22.65 ± 11.55	23.44 ± 12.26	
Treatment effect; LS mean ± SE (95%CI)	− 1.32 ± 0.70 (− 2.72, 0.74)	− 0.49 ± 0.72 (− 1.91, 0.92)	0.413^a^
*PDQ-8 score*			
Baseline; mean ± SD	8.70 ± 3.77	8.93 ± 4.26	
Week 12; mean ± SD	6.93 ± 3.50	7.34 ± 3.86	
Treatment effect; LS mean ± SE (95%CI)	− 1.80 ± 0.28 (− 2.37, − 1.25)	− 1.54 ± 0.29 (− 2.11, − 0.98)	0.514^a^
*GDS-15 score*			
Baseline; mean ± SD	6.36 ± 3.00	6.99 ± 3.08	
Week 12; mean ± SD	5.10 ± 3.00	5.83 ± 3.11	
Treatment effect; LS mean ± SE (95%CI)	− 1.37 ± 0.25 (− 1.86, − 0.88)	− 1.05 ± 0.25 (− 1.55, − 0.55)	0.373^a^
*PDSS-2 score*			
Baseline; mean ± SD	20.49 ± 13.81	21.70 ± 15.44	
Week 12; mean ± SD	13.92 ± 12.28	18.43 ± 14.95	
Treatment effect; LS mean ± SE (95%CI)	− 6.70 ± 0.79 (− 8.25, − 5.15)	− 3.15 ± 0.80 (− 4.72, − 1.57)	0.002^a*^
Clinical remission; n (%)	41 (46.1%)	27 (31.0%)	0.041^b*^
Responder; n (%)	31 (34.8%)	16 (18.4%)	0.014^b*^

Parametric variables are presented as mean ± SD, and categorical variables are presented as n (%)

HAMD, Hamilton Depression Scale; UPDRS-III, Unified Parkinson Disease Rating Scale part III; PDQ-8, Parkinson’s Patient Quality of Life Scale; GDS-15, Geriatric Depression Scale; PDSS-2, Parkinson’s Disease Patient Sleep Quality Scale; SD, standard deviation, SE, standard error; CI, confidence interval; LS, least squares

^*^p < 0.05

^a^Repeated-measures ANOVA

^b^Chi-squared test

Secondary efficacy outcomes are listed in Table 2. PDSS-2 scores were also significantly improved in the ZPG group than in the placebo group (LS mean difference ± SE, − 3.56 ± 1.12; 95% CI, − 5.77, − 1.35; p = 0.002). However, no significant difference was observed for UPDRS Part III, PDQ-8, or GDS-15 at week 12.

### Safety and tolerability

Treatment-emergent AEs are listed in Table 3. Two subjects in the ZPG group discontinued the study because of AEs, compared with three in the placebo group. Nausea led to discontinuation in three patients (n = 2 in the ZPG group and n = 1 in the placebo group), and abdominal pain led to discontinuation in 2 patients (one in the ZPG group and one in the placebo group). No serious AEs in either group were observed.

**Table 3:** Summary of treatment-emergent AEs (safety population)

Event	Zishen Pingchan (n = 99)	Placebo (n = 100)
Nausea	2 (2.0%)	2 (2.0%)
Diarrhea	1 (1.0%)	0
Abdominal pain	1 (1.0%)	0
Vomiting	1 (1.0%)	0
Stomach pain	1 (1.0%)	2 (2.0%)
Facial swelling	1 (1.0%)	0
Constipation	0	2 (2.0%)
Abnormal liver function	0	2 (2.0%)
Muscle aches	0	1 (1.0%)
Total	7 (7.1%)	9 (9.0%)

Data are reported as n (%)

AE, adverse event

## Discussion

In this perspective, randomized, double-blind trial, ZPG provided significant improvements in HAM-D scores and PDSS-2 scores when added to PPX therapy. The ZPG group showed better safety and tolerance without serious AEs compared with the placebo-controlled group. Thus, improvements were achieved without compromising tolerability.

After 12 weeks add-on treatment with ZPG, patients in the ZPG group showed a significant improvement of depression compared with those in the placebo group, a result consistent with previous studies [10]. The etiology of dPD is complicated. Some studies suggest that impaired monoaminergic neurotransmission contributes to dPD [15, 16], and ZPG may suppress the over-activation of the c-Jun N-terminal protein kinase (JNK) pathway in the substantia nigra, alleviate the inflammatory response in nigral cells, protect the dopaminergic neuron and finally improve depression [17]. Besides, the regulation of dopamine receptors is considered as playing an important role in the pathogenesis of depression [18–22]. PPX improves depressive symptoms by activating dopamine D3 receptor in an animal experiment [23] through its role as a selective dopamine receptor agonist[24]. In addition, dopamine D2 receptor is closely related to schizophrenia, bipolar disorder, and severe depression [25]. According to a previous report, ZPG up regulates the gene expression of dopamine D2 receptors in rats [26] and selective D2 dopamine receptor agonists relieve depression in stressed rats by up-regulating tyrosine hydroxylase [27]. Since D3 and D2 dopamine receptors are 75% homologous in their transmembrane domains, this indicates that their functions tend to be synergistic. D3 dopamine receptors exert some modulatory effect on many of the functions generally attributed to D2 dopamine receptors [22, 28]. Therefore, ZPG combined with PPX was a good method for treating dPD.

Since PD and depression were both placebo-prone diseases, the potential placebo effect was considered in this study [29]. The scores of HAM-D, GDS-15, UPDRS-III, PDQ-8, and PDSS-2 in the placebo group improved compared with the baseline at 12 weeks, indicating that the placebo effect could improve the motor and non-motor symptoms of PD patients, although this effect had individual differences [29]. Despite the placebo effect, both the remission rate and responder rate in the ZPG group were significantly higher than those in the placebo group. This result showed that although it was not possible to identify which patients may benefit from the potential placebo effect, in this double-blind, placebo-control trial, ZPG showed a significantly better therapeutic effect than the placebo group, proving the value of ZPG in clinical application. However, no statistically significant difference in the mean change of HAM-D between the two groups was observed at week 6. The probably reason was that, unlike the tablets in the previous placebo-controlled studies [7, 30], the granular placebo used in this work had more similar characteristics to ZPG, resulting in a stronger placebo effect that persisted throughout the trial. Our hypothesis was that ZPG took effect quickly in the first 2 weeks, thus showing a slight advantage in the second week. No significant difference between the two groups was observed at week 6 because the placebo effect was still working. Then, since in the final visit the effects of ZPG obviously outweighed the placebo effect, a significant improvement in depression was observed.

Among secondary outcomes, a significant improvement in sleep quality was observed, consistent with previous studies [10, 31]. This might be due to the presence of Yuanzhi (Yuǎn zhì) and Shichangpu (Shí chāng pú) in the prescription. The traditional Chinese medicine Polygala has sleep-promoting, anti-inflammatory, and sedative effects, which may be achieved through the serotonergic system and the gamma-aminobutyric acid system [32]. Besides, Acori Tatarinowii Rhizoma (Shí chāng pú) is also a commonly used traditional Chinese medicine in the treatment of insomnia and epilepsy [33]. It was worth noting that, considering the complexity of PD sleep disorders, it is still necessary to further explore the mechanism used by ZPG to improve sleep in PD. However, no significant improvements in motor symptoms and quality of life were observed, probably because although ZPG can up-regulate the expression of dopamine receptors, it is not a dopaminergic drug after all, which may limit its effect on motor symptoms. In addition, the quality of life did not improve, probably because PDQ-8 contains too many dimensions including Mobility, Activity of daily living, Emotional well-being, Stigma, Social support, Cognitions, Communication and Bodily discomfort. Improvement in depression involves one or two of these dimensions and therefore may not well reflect the improvement in quality of life.

ZPG had good safety and tolerance, both the ZPG group and the placebo group had a relatively low incidence of AEs (7.1% *vs* 9%), and both groups had mild to moderate AEs. Three patients in the ZPG group abandoned the trial after AEs, while only two patients in the placebo group abandoned the trial after AEs, while the remaining nine patients with AEs completed the trial, with a total withdrawal rate of 2.5%. These AEs disappeared within a few weeks after drug withdrawal, indicating that ZPG was well tolerated and safe.

The Strength of this study included the following aspects: (1) This study was a multicenter, randomized, double-blind placebo-controlled trial with a good design and relatively large sample size. (2) the composition of the Zishen Pingchan recipe was modified by adding two traditional Chinese medicines, Zhimu and Baihe because some studies showed that Zhimu-baihe Decoction significantly improves the symptoms of depression in PD patients [34, 35]. This change may increase the efficacy of ZPG and exert more benefits in dPD patients.

Some limitations in our study also exist, which should be considered. (1) Patients with severe depressive symptoms were not recruited in this study, which might have limited the potential scale of the treatment effect and the generalizability. Our plan is to recruit more dPD patients, including those with severe depression, in future studies to further expand the universality of the results of this study. (2) The Han population was the only one included, and the results need to be further verified by high-quality double-blind, placebo-controlled trials with larger sample size and longer follow-up of different ethnic groups. (3) The results of sleep disorders and mental disorders in this study are subjectively self-reported, which may lead to evaluation bias. Therefore, polysomnography and functional magnetic resonance imaging should be used in the future. (4) Our study included some young PD patients (the age at the onset was less than 45 years) [36] but the patients’ genes were not tested. Since some mutations may cause a different response to drugs, it is necessary to test the patients genes in the future.

## Conclusion

In conclusion, this randomized, double-blind, placebo-controlled trial demonstrated the addition of ZPG to PPX further improved the depression symptoms and sleep quality of dPD patients with good tolerability, providing an important evidence-based medicine basis, which was worthy of further promotion in clinical practice.

## Data Availability

The datasets used in this study are available from the corresponding authors upon reasonable request.

## Abbreviations

PD: Parkinson’s disease
dPD: Depression in the Parkinson’s disease
ZPG: Zishen Pingchan Granules
PPX: Pramipexole
HAM-D: Hamilton Depression Scale
GDS-15: Geriatric Depression Scale
UPDRS III: Unified Parkinson's disease rating scale Part III
PDQ-8: Parkinson's quality of life scale
PDSS-2: Parkinson's disease sleep scale
AEs: Adverse events
MMSE: Mini-mental State Examination
mITT: Modified intent-to-treat
LOCF: Last observation carried forward
SD: Standard deviation
SE: Standard error
CI: Confidence interval
LS: Least squares
JNK: C-Jun N-terminal protein kinase
LED: Levodopa equivalent dose
